# Impact of heterozygote CFTR Mutations in COPD patients with Chronic Bronchitis

**DOI:** 10.1186/1465-9921-15-18

**Published:** 2014-02-11

**Authors:** S Vamsee Raju, Jody H Tate, Sandra KG Peacock, Ping Fang, Robert A Oster, Mark T Dransfield, Steven M Rowe

**Affiliations:** 1 Department of Medicine, University of Alabama at Birmingham, MCLM 706 1918 University Blvd., Birmingham, AL, USA; 2 Department of Pediatrics, University of Alabama at Birmingham, Birmingham, AL, USA; 3 Departments of Cell Developmental and Integrative Biology, University of Alabama at Birmingham, Birmingham, AL, USA; 4 The Gregory Fleming James Cystic Fibrosis Research Center, University of Alabama at Birmingham, Birmingham, AL, USA; 5 UAB Lung Health Center, University of Alabama at Birmingham, Birmingham, AL, USA; 6 Medical Genetics Laboratories, Baylor College of Medicine, Houston, TX, USA

## Abstract

**Background:**

Cigarette smoking causes Chronic Obstructive Pulmonary Disease (COPD), the 3rd leading cause of death in the U.S. CFTR ion transport dysfunction has been implicated in COPD pathogenesis, and is associated with chronic bronchitis. However, susceptibility to smoke induced lung injury is variable and the underlying genetic contributors remain unclear. We hypothesized that presence of CFTR mutation heterozygosity may alter susceptibility to cigarette smoke induced CFTR dysfunction. Consequently, COPD patients with chronic bronchitis may have a higher rate of CFTR mutations compared to the general population.

**Methods:**

Primary human bronchial epithelial cells derived from F508del CFTR heterozygotes and mice with (CFTR+/-) and without (CFTR+/+) CFTR heterozygosity were exposed to whole cigarette smoke (WCS); CFTR-dependent ion transport was assessed by Ussing chamber electrophysiology and nasal potential difference measurements, respectively. Caucasians with COPD and chronic bronchitis, age 40 to 80 with FEV_1_/FVC < 0.70 and FEV_1_ < 60% predicted, were selected for genetic analysis from participants in the NIH COPD Clinical Research Network’s Azithromycin for Prevention of Exacerbations of COPD in comparison to 32,900 Caucasian women who underwent prenatal genetic testing. Genetic analysis involved an allele-specific genotyping of 89 CFTR mutations.

**Results:**

Exposure to WCS caused a pronounced reduction in CFTR activity in both CFTR (+/+) cells and F508del CFTR (+/-) cells; however, neither the degree of decrement (44.7% wild-type vs. 53.5% F508del heterozygous, P = NS) nor the residual CFTR activity were altered by CFTR heterozygosity. Similarly, WCS caused a marked reduction in CFTR activity measured by NPD in both wild type and CFTR heterozygous mice, but the severity of decrement (91.1% wild type vs. 47.7% CF heterozygous, P = NS) and the residual activity were not significantly affected by CFTR genetic status. Five of 127 (3.9%) COPD patients with chronic bronchitis were heterozygous for CFTR mutations which was not significantly different from controls (4.5%) (P = NS).

**Conclusions:**

The magnitude of WCS induced reductions in CFTR activity was not affected by the presence of CFTR mutation heterozygosity. CFTR mutations do not increase the risk of COPD with chronic bronchitis. CFTR dysfunction due to smoking is primarily an acquired phenomenon and is not affected by the presence of congenital CFTR mutations.

## Introduction

Chronic obstructive pulmonary disease (COPD) is the 3rd leading cause of death in the U.S. and mortality is increasing [1]. An improved understanding of COPD pathogenesis, and the genes that contribute to its progression, are needed to develop therapeutic approaches to the disease [2]. Susceptibility to lung injury from cigarette smoking is highly variable, with only ~15-20% of at risk individuals developing clinically significant COPD. While the presence of the Z allele of the alpha-1-antitrypsin (AAT) gene has been shown to increase the risk for COPD [3,4], it only accounts for a small fraction of cases. GWAS studies have not yet identified genetic contributors with a firm link to disease pathogenesis [5,6]. Complicating this further is the fact that the phenotypic expression of COPD is highly variable, with some individuals developing disease dominated by emphysema while others exhibit chronic bronchitis or both [7,8] which likely reflects the contribution of multiple pathologic mechanisms.

There are more than 10 million Americans who are asymptomatic carriers of at least one CFTR mutation [9]. A number of disorders are associated with mild/variable CFTR mutations that cause intermediate phenotypic expression. For example, 30-50% of patients with idiopathic pancreatitis are heterozygotes for CFTR mutations [10,11], and a similar association has been made for congenital bilateral absence of the vas deferens [12] and allergic pulmonary aspergillosis [13]. We have previously reported that smokers with COPD have decreased CFTR function in both the upper [14] and lower airways [15], confirming prior studies in healthy smokers [16,17] and suggesting that CFTR dysfunction may also play a role in the pathogenesis of COPD. CFTR dysfunction was also found to be associated with chronic bronchitis symptoms and dyspnea [14,15], indicating CFTR abnormality may be particularly important towards causing mucus retention, and supports in vitro studies indicating reduced airway surface liquid depth [14,17] and delayed mucociliary transport [14,18] caused by cigarette smoke exposure. CFTR dysfunction has also been observed in mice exposed to whole cigarette smoke [19]. Since, a surprisingly large percentage of COPD patients have recently been found to have bronchiectasis by high resolution CT [20] and because chronic bronchitis shares many pathologic similarities with CF, it follows that genetic CFTR abnormalities may increase the prevalence of chronic bronchitis in smokers.

A previous study identified a moderate association between F508del CFTR mutations and in individuals with chronic bronchitis and sweat chloride levels of 60 mmol/L or higher [21]. Other studies demonstrating an association have been small and have not examined the frequency of CFTR mutations in COPD subtypes [22-24]. An association between common CFTR mutations and chronic bronchitis was not observed by Entzian et al. [25]. Similarly, earlier studies from Germany and Japan failed to detect an association between CFTR mutations and COPD [26,27], although neither focused on individuals with chronic bronchitis.

In this study, we hypothesized CFTR mutation heterozygosity would increase the susceptibility to cigarette smoke induced CFTR dysfunction. To our surprise, in vitro and in vivo studies revealed an absence of a gene-dose effect between cigarette smoking and CFTR dysfunction. This was further supported by the absence of increased CFTR mutation frequency in chronic bronchitis patients when compared to the general population. These findings indicate that CFTR dysfunction due to smoking is primarily an acquired phenomenon, and that CFTR mutations do not significantly increase the prevalence of acquired CFTR dysfunction induced chronic bronchitis.

## Methods

### In vitro experiments with primary human airway epithelial cells

UAB Institutional Review Board approved the use of human cells. Primary human bronchial epithelial cells (HBE) were obtained from lung explants. Genetic analysis was performed to identify cells with expression of wild type CFTR (CFTR +/+) and those heterozygous for non-functional (i.e. F508del) CFTR mutations (CFTR +/-) following previously described methods [28]. After expanding isolated HBE cells, first or second passage cells were seeded on permeable support filters (Corning, Lowell, MA) coated with NIH 3 T3 fibroblast conditioned media. HBE cells were grown in differentiating media for 6 weeks until terminally differentiated, as previously described [14].

HBE cells were exposed to WCS from one 3R4F research cigarette (University of Kentucky, Lexington, KY) for 10 min. WCS was generated via an automated cigarette smoke generator (Scireq InExpose model, Toronto, Canada) at 1 puff/min at a flow rate of 3 L/min, as previously described [19]. Controls cells were similarly exposed to room air.

CFTR-dependent short circuit current was measured in Ussing chambers under voltage clamp conditions using MC8 voltage clamps and P2300 Ussing chambers (Physiologic Instruments, San Diego, CA) [14]. CFTR activity was measured by the change in Isc upon stimulation with forskolin (10 μM) in the setting of amiloride (100 μM). A Cl^-^ secretory gradient was used, where indicated; CFTR_Inh_-172 (10 μM) was used to confirm CFTR dependence [29].

### In vivo cigarette smoke exposure

Animal protocols were approved by the UAB Institutional Animal Care and Use Committee. Age and sex matched congenic C57BL/6 J mice expressing wild type CFTR (CFTR +/+) or heterozygous for CFTR knock out (CFTR +/-, C57BL/6 J Cftrtm1Unc/J) were used. Mice were exposed in whole-body chambers (28″ × 19″ × 15″) to mainstream cigarette smoke (200 μg/l of total particulate matter, 35-ml puffs of 2-s duration at a rate of 3 L/sec each minute for 20 min) from 4 3R4F reference cigarettes (Univ. of Kentucky, Lexington, KY) twice daily for 2 weeks using an automated cigarette smoking apparatus (SCIREQ, InExpose model, Toronto, Canada). Control mice were exposed to room air under similar conditions [19,30].

### Measurement of murine CFTR activity

Murine CFTR function was assessed by nasal potential difference measurements*.* Under anesthesia, CFTR-dependent anion transport was measured in the murine nasal epithelium as a change in potential difference following perfusion with chloride free forskolin (10 μM) in the setting of amiloride (100 μM), as previously described [19,29].

Mice were euthanized and tracheas were harvested by clean surgical techniques. Tracheal epithelia were mounted and tested as full-thickness tissue. Isc was measured under voltage clamp conditions as performed in cells using P2307 Ussing chamber sliders. Mounted tissues were bathed on both sides with identical Ringers solutions gassed with 95% O_2_:5% CO_2_ and then treated with amiloride (100 μM) followed by the CFTR agonists forskolin (10 μM) and IBMX (100 μM); bumetanide (10 μM) and glybenclamide (100 μM) were added to the mucosal solution at the end of experiments to block CFTR-dependent Isc. Results are expressed as the change in Isc with agonist stimulation [19,31].

### Genetic analysis of chronic bronchitis

We performed a case control study to detect the prevalence of CFTR mutations in COPD patients with symptoms of chronic bronchitis. The UAB IRB approved use of clinical specimens for the research study. COPD cases were selected from participants in the NIH COPD Clinical Research Network’s Azithormycin in COPD Study who had blood stored for genetic analysis [32]. Cases were non-Hispanic Caucasians, age 40 to 80 with FEV_1_/FVC < 0.70 and FEV_1_ < 60% predicted, and chronic bronchitis defined by productive cough “most days a week” or “several days of the week” on the St. George Respiratory Questionnaire (SGRQ). The control population was 32,900 Caucasian women who had CFTR analyzed for prenatal genetic testing, N = 32,900. No further demographic characteristics are available for this population.

DNA testing was performed by the Baylor College of Medicine Genetics Laboratory using a CFTR related disorders mutation panel. This is an allele-specific genotyping technique for 89 mutations (Table 1) performed by MALDI-TOF mass spectrometry and includes testing for mutations common in human populations and reflex testing of the 5 T allele. The method has an 88% CFTR mutation detection rate in the non-Hispanic Caucasian population. Additional data collected on COPD cases included age, sex, smoking status, smoking intensity (pack-years), FEV_1_% predicted, and the number of COPD exacerbations requiring treatment in the year prior to enrollment in the azithromycin trial. The sample size of 127 patients had 80% power to detect a mutation rate in COPD patients of 10.5% as compared to a presumed frequency of 4.5% in controls with an alpha of 0.05.

**Table 1:** List of CFTR mutations analyzed

F508del	R117H	1717-1G > A	R117C
G85E	R334W	1898 + 1G > A	Y122X
A455E	R347P	2184delA	G178R
I507del	R553X	2789 + 5G > A	G314E
G542X	R560T	3120 + 1G > A	G330X
G551D	W1282X	3659delC	R347H
N1303K	621 + 1G > T	K710X	406-1G > A
R1162X	711 + 1G > T	E60X	G480C
R1066C	W1089X	V520F	A559T
S1196X	Q1238X	S1251N	S1255X
663delT	935delA	1161delC	1288insTA
2184insA	2307insA	2711delT	2869insG
R709X	R764X	R1158X	574delA
Q493X	1898 + 5G > T	3905insT	I506T
3849 + 10kbC > T	712-1G > T	Q98R	Q552X
S549N	1078delT	H199Y	444delA
S549R (T > G)	2143delT	P205S	2043delG
1811 + 1.6kbA > G	3272-26A > G	L206W	3791delC
Y1092X (C > G)	3199del6	F508C	2108delA
Y1092X (C > A)	D1152H	V520I	3667del4
394delTT	3876delA	M1101K	1677delTA
W1098X (TGA)	1812-1G > A	4016insT	1609delCA
3171delC			

### Statistics

For in vitro and in vivo studies, descriptive statistics (mean, SD, and SEM) were compared using Student’s *t*-test or ANOVA, as appropriate. Post-hoc tests for multiple comparisons were calculated using Fisher’s least significant difference. Population statistics were performed using SAS (IBM, Armonk, NY). Chi-square or Fischer’s exact test used for inferential comparisons. All statistical tests were two-sided and were performed at a 5% significance level (i.e., α = 0.05) using GraphPad Prism (La Jolla, CA). Error bars designate SEM unless indicated otherwise.

## Results

### Effect of CFTR mutation heterozygosity on smoke-induced CFTR dysfunction in vitro

**Figure 1:**
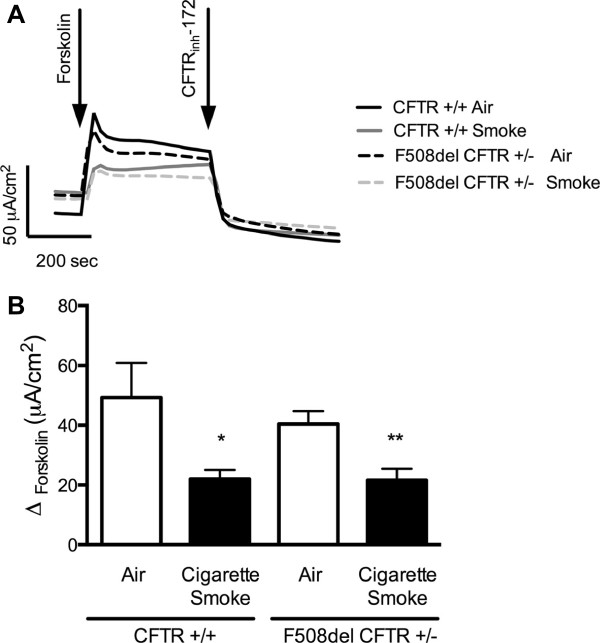
**Effect of CFTR mutation heterozygosity on smoke-induced CFTR dysfunction *in vitro*.** Well differentiated primary human bronchial epithelial cells (HBE) cells were isolated from healthy non-smokers and non-CF individuals and expressing either wild type CFTR (CFTR +/+) or heterozygous F508del CFTR mutation (CFTR +/-). These cells were exposed to cigarette smoke generated from 1 cigarette for 10 min and CFTR function measured in Ussing chambers under voltage clamp conditions. **A)** Representative tracings of forskolin stimulated CFTR ion transport in HBE cells in vitro. Addition of forskolin (20 μM) and CFTR_inh_172 (10 μM) are indicated. **B)** Forskolin-stimulated CFTR-dependent anion transport is summarized, N = 6-8 *P < 0.05, **P < 0.005.

Since smokers with and without COPD exhibit reduced CFTR mediated anion transport [14-17,19], and this is associated with chronic bronchitis [14,15,19], we hypothesized that acquired CFTR dysfunction may also be influenced by the presence of congenital CFTR mutations. As a test of this hypothesis, we exposed primary HBE cells heterozygous for the F508del CFTR mutation to whole cigarette smoke and compared this to the degree of CFTR decrement observed in HBE cells without CFTR mutations (e.g. wild-type expressing monolayers). As expected based on genotype-phenotype correlations in the disease [33], HBE cells derived from a F508del CFTR heterozygote had slightly lower CFTR activity at baseline than wild type monolayers as measured by response to forskolin stimulation (49.3 ± 11.5 μA/cm^2^ in CFTR (+/+) vs. 40.5 ± 5.3 μA/cm^2^ in CFTR (+/-), although this was not statistically significant (Figure 1A,B). WCS caused a pronounced reduction (27.3 ± 3.1 μA/cm^2^) in CFTR activity in CFTR (+/+) monolayers (P < 0.05), consistent with prior studies using WCS [17,19,30,34,35] and CSE [14,36]. WCS also caused a significant decrement in CFTR (+/-) cells (18.8 ± 2.4 μA/cm^2^; P < 0.005). Both the relative decrement (44.7% vs. 53.5%, P < 0.93) and the degree of residual CFTR activity in CFTR (+/+) and CFTR (+/-) cells following WCS exposure (22.1 ± 3.0 μA/cm^2^ in CFTR +/+ vs. 21.6 ± 3.8 μA/cm^2^ in CFTR +/-) was indistinguishable, indicating the presence of a single F508del CFTR mutation did not meaningfully alter the CFTR decrement from environmental exposure to WCS in vitro.

### Effect of CFTR mutation heterozygosity on smoke-induced CFTR dysfunction in vivo

**Figure 2:**
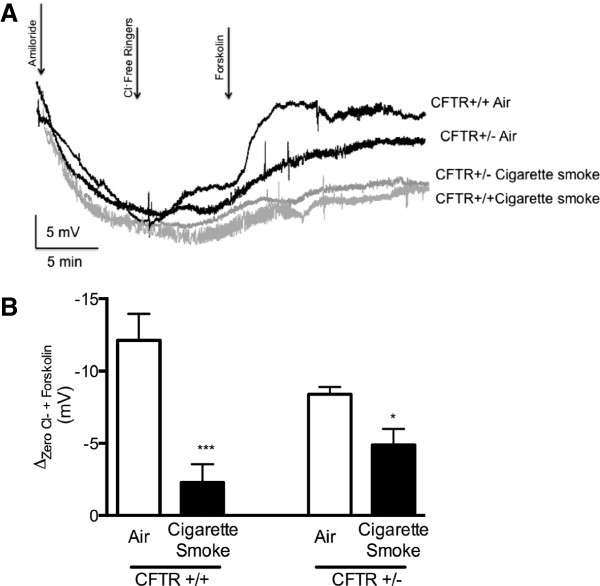
**Effect of CFTR mutation heterozygosity on smoke-induced CFTR dysfunction *in vivo*.** C57BL/6 J mice expressing wild type CFTR (CFTR +/+) or heterozygous CFTR (CFTR +/-) were exposed to whole cigarette smoke (4 cigarettes, twice daily) or room air control in whole body chambers for 2 weeks prior to CFTR functional estimation with nasal potential difference (NPD). **A)** Representative tracings of NPD measured in CFTR +/+ or CFTR +/- mice exposed to either cigarette smoke or air control. Initiation of amiloride (100 μM), Cl^-^ free ringers and Cl^-^ free ringers plus forskolin (20 μM) are indicated. **B)** Mean forskolin stimulated change in nasal potential difference, an indicator of CFTR mediated cl^-^ transport. N = 8/condition, *P < 0.05, **P < 0.005.

**Figure 3:**
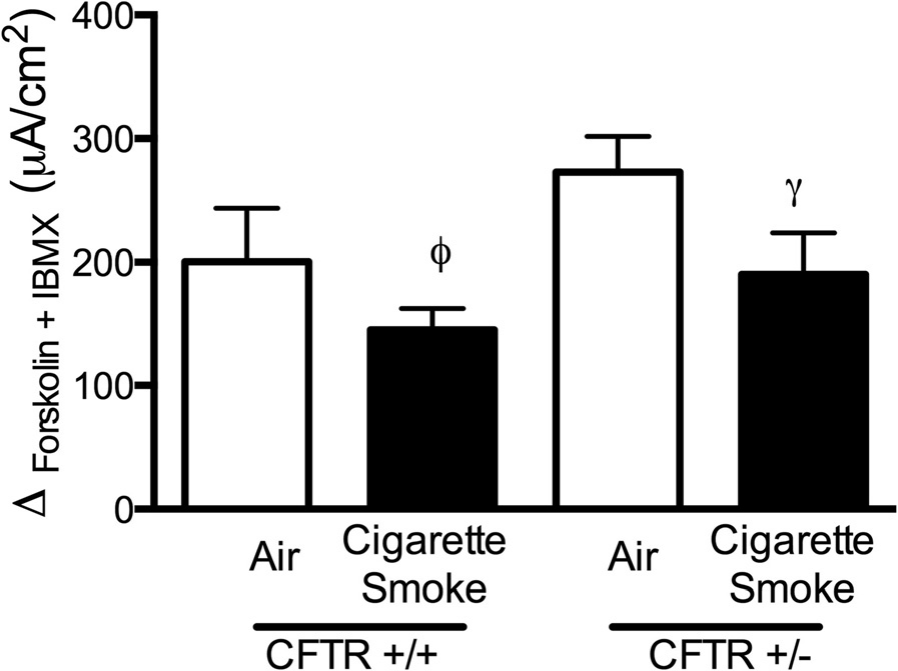
**Effect of CFTR mutation heterozygosity on smoke-induced CFTR dysfunction in murine trachea.** C57BL/6 J mice expressing wild type CFTR (CFTR +/+) or heterozygous CFTR knockout (CFTR +/-) were exposed to whole cigarette smoke (4 cigarettes, twice daily) or room air control in whole body chambers for 2 weeks prior to CFTR functional estimation in trachea ex-vivo by Ussing chamber electrophysiology. CFTR activity was estimated as cAMP-dependent change in tracheal short circuit current (Isc) by stimulating with forskolin (20 μM) plus IBMX (100 μM). N = 8/condition, ϕ = 0.25 and, γ = 0.08.

To better understand findings in human bronchial epithelial cells and provide definitive evidence regarding the contribution of heterozygous CFTR mutations to cigarette smoke induced CFTR dysfunction, we next used an animal model of cigarette smoke exposure. Congenic wild type (CFTR +/+) and heterozygote (CFTR +/-, C57BL/6 J Cftrtm1Unc/J) CF57BL/6 J mice were exposed in whole body chambers to cigarette smoke twice daily for 2 weeks, and then CFTR activity was measured in the respiratory tract by nasal potential difference (NPD) and short circuit current analysis of freshly excised trachea. As expected based on genotype-phenotype correlations in the disease, and observed in prior NPD studies in humans [33], NPD in heterozygote CFTR (+/-) mice had slightly lower CFTR activity when compared to the wild type mice (-12.1 ± 1.8 mV in CFTR (+/+) vs. -8.7 ± 0.6 mV in CFTR (+/-)), although this was not statistically significant (P < 0.14, Figure 2). Cigarette smoke exposure caused a significant decrease in CFTR-mediated ion transport in the nasal airway that was similar in severity to the observations in HBE cells (Figure 2). WCS caused a pronounced reduction (-11.1 mV) in CFTR activity in CFTR (+/+) mice (P < 0.005), a finding recapitulated in CFTR (+/-) mice (-4.1 mV; P < 0.05). Although the absolute reduction in CFTR activity was less prominent in heterozygous mice, the residual cAMP dependent CFTR activity in CFTR (+/+) and CFTR (+/-) mice following WCS exposure were no different and favored heterozygous animals (-0.99 ± 2.07 mV in CFTR (+/+) vs. -4.57 ± 2.05 mV in CFTR (+/-), P = 0.26). Analysis of short-circuit current of excised trachea demonstrated a similar trend, with reduced CFTR dependent ion transport observed in both wild type and heterozygous trachea following WCS exposure that was not meaningfully different (Figure 3). The lack of statistically significant reduction in trachea CFTR activity following a 2-week exposure to WCS is consistent with previously reported data since this tissue exhibits time-dependent decrements [19]. Taken together, these data provide evidence that heterozygosity imposed by the presence of one CFTR mutation does not increase the susceptibility or magnitude of CFTR dysfunction following cigarette smoke exposure.

### Prevalence of CFTR mutations in patients with COPD and chronic bronchitis

The lack of the expected increase in susceptibility to cigarette smoke induced CFTR dysfunction due to the absence of one copy of CFTR prompted us to determine the prevalence of CFTR mutations among COPD patients with chronic bronchitis in comparison to the general Caucasian population. We evaluated 127 individuals with COPD and chronic bronchitis who participated in the Azithromycin in COPD Study sponsored by the COPD Clinical Research Network [37]. To enrich the population for those likely to exhibit CFTR abnormality, and assure best matching with our control group, we restricted the analysis to individuals with COPD, chronic bronchitis and of Caucasian descent. As shown in Table 2, the population had a slight male predominance, and 25% were current smokers. The mean FEV_1_% predicted reflected a moderate to severely ill population. There was a high frequency of health care utilization for respiratory symptoms and pulmonary exacerbations. Five of the 127 subjects (3.9%) had detectable CFTR mutations based on a genetic panel containing 89 mutations. The frequency of CFTR mutations among those with COPD and chronic bronchitis was no different than the mutation rate seen in the control population (1356 of 32900, 4.1%; P = NS; Table 3). Limiting the analysis to the F508del CFTR mutation, only 2 (1.5%) of cases were positive, which was again no different than controls (2.7%; P = NS).

**Table 2:** Characteristics of COPD study subjects undergoing CFTR genetic analysis

**Characteristics**	
Age (years)	62
Male (%)	65.5
Current smokers (%)	25
Average pack/years	64
Hospitalizations or emergency department visits for respiratory symptoms in the previous year (%)	48
Required antibiotics for chest infections in previous 3 months (%)	41
Required oral steroids for respiratory symptoms in previous 3 months (%)	37
Average FEV_1_ (% predicted)	36

FEV_1_- Forced Expiratory Volume in one second.

**Table 3:** Prevalence of CFTR mutations in Caucasian COPD and control subjects

**Study population**	**With mutation**	**Without mutation**	**P Value**
**COPD N = 127**			
Any mutation	5 (3.9%)	122 (96.1%)	0.92
F508del	2 (1.5%)	125 (98.5%)	0.63
Non-F508del	3 (2.4%)	124 (97.6%)	0.63
**Controls N = 32,900**			
Any mutation	1,356 (4.1%)	31,544 (95.9%)	
F508del	880 (2.7%)	32,020 (97.3%)	
Non-F508del	476 (1.4%)	32,424 (98.6%)	

P-values presented are Chi-square analyses compared to general Caucasian population.

## Discussion

A number of studies have shown that cigarette smoke causes acquired CFTR dysfunction in smokers with and without COPD [14,15,17,19,30,38]. Here, we evaluated the effect CFTR heterozygosity on this pathway using in vitro and in vivo models of smoke exposure. As expected, cigarette smoke exposure reduced CFTR function in mice and cells, consistent with prior literature [25,26]. However, CFTR heterozygosity did not cause a functional disadvantage in either mice or primary human epithelia. At baseline mice and cells with CFTR (+/-) expression had slightly lower ion transport function compared to their wild type controls (~90% of WT). However, following smoke exposure, each reached similar levels (~40% of WT). Hence, the presence of a protective 2nd normal CFTR allele was not sufficient to overcome the functional decrement caused by smoke. These data we provide the first experimental evidence that acquired CFTR dysfunction from environmental exposure to cigarette smoke or other COPD related pathology is the principle contributor to the defective ion transport phenotype [15,19] and is not substantially affected by the presence of CFTR mutation heterozygosity. The degree of CFTR decrement observed following smoke exposure and due to CFTR mutation heterozygosity alone were each consistent with prior reports in carriers [33], smokers [14,15], cells [17,30,36], and mice [19] exposed to cigarette smoke and CF, lending further confidence to our findings.

To confirm findings in the laboratory, we also investigated whether genetic heterozygosity for CFTR mutations contributes to the prevalence of COPD with chronic bronchitis in cigarette smokers. We found that CFTR mutations are not more frequent in COPD patients with moderate to severe obstruction and chronic bronchitis symptoms compared to a control population of Caucasian individuals. Despite clear evidence that cigarette smoke reduces CFTR mediated anion transport, these results suggest that congenital CFTR mutations do not contribute to a genetic predisposition to the prevalence of COPD and chronic bronchitis. Further, our in vitro and in vivo data provide an explanation for the absence of enhanced susceptibility to cigarette smoke induced chronic bronchitis in individuals heterozygous for CFTR mutations.

Unlike Gervais et al., we did not demonstrate an increased association in the F508del CFTR allele and chronic bronchitis, however this study observed the association in individuals with elevated sweat chloride in an era before comprehensive CFTR genetic analysis, potentially resulting in the inclusion of patients with a mild form of cystic fibrosis due to two unrecognized CFTR mutations; smoking related COPD was also not specified in this report [21]. Our findings also contrast to Kostuch et al. who reported a slight increase (5 out of 32) in incidence of F508del mutations in chronic bronchitis patients in Poland. Although the authors attempted to enrich subjects for chronic bronchitis, the criterion used in that study was based on persistent chronic effective cough alone, and did not include any other clinical parameters such as a history of smoking. Similarly, we did not observe any increase in the incidence of M470V or R75Q as reported earlier in COPD patients from Serbia [22,23]. These studies were based on small subject groups (<20) and were based on limited clinical phenotyping. Further, the M470V polymorphism is no longer thought to contribute to CF disease [39]. Differences in the baseline prevalence of these less common CFTR mutations due to genetic founder effects may also have contributed to the disparate results. Despite these distinctions, the conclusion reported in this manuscript are based on a more complete clinical evaluation and are in agreement with conclusions drawn from large unbiased genetic approaches, including recent GWAS analyses, where no apparent increase in CFTR mutations was observed [40].

The lack of an association with the prevalence of chronic bronchitis stand in contrast to studies in asthma where in a Swedish cohort of asthmatics, F508del CFTR heterozygosity was significantly more prevalent when compared to the control population. Further, asthmatic individuals heterozygous for CFTR had decreased pulmonary function and airway obstruction in comparison to those without mutant CFTR alleles [41]. Similarly, CFTR mutations have been reported in increased frequency among patients with non-CF chronic rhinosinusitis [35]. This may be due to differences in the biology of COPD as compared to these disorders, or the size of our study.

Our study has important limitations. Since, our analyses were focused on one sub-phenotype of COPD (i.e. chronic bronchitis), it would be beneficial to conduct additional CFTR genetic analysis in a larger cohort of subjects to rule out CFTR as a contributor. For example, there are over 1900 potential disease-causing mutations in CFTR [42] and our analysis was limited to the top 89. Even though our analyses included 88% of alleles found in Caucasians, CFTR sequencing may detect an association not identified by our analyses. Furthermore, the same principle could reduce the sensitivity of GWAS analyses intended to detect an association with CFTR mutations, since SNPs could be spread over many locations along the CFTR gene, depending on the allele. Our study was performed in a modest sized cohort and would be strengthened by a targeted approach in a much larger sample. CFTR genetics could also influence the severity of the disease, rather than prevalence, and was not assessed by this analysis. While our animal studies recapitulated that observed humans, they are not useful to evaluate chronic bronchitis, since neither mice deficient in the CF gene or chronically exposed to cigarette smoke develop mucus obstruction or retention. Larger animal models may be needed for studies of this sort.

## Conclusion

In total, the experimental data indicate why smokers with CFTR mutations may not exhibit a genetic predisposition to develop chronic bronchitis or a gene dose effect, even if chronic bronchitis symptoms are partially mediated by abnormal CFTR function. Our data suggest that effects of cigarette smoke on CFTR function acquired by environmental exposure are far more influential to clinical phenotype than inherited defects due to mutations in CFTR. These results may inform appropriate patient selection for trials evaluating CFTR modulators or ion channel agonists for the treatment of COPD [14,30].

## Abbreviations

cAMP: Cyclic adenosine monophosphate; CF: Cystic fibrosis; CFTR: Cystic fibrosis transmembrane conductance regulator; COPD: Chronic obstructive pulmonary disease; WCS: Whole cigarette smoke; CSE: Cigarette smoke extract; HBE: Human bronchial epithelial; GWAS: Genome wide association studies; Isc: Short circuit current; LAPD: Lower airway potential difference; NPD: Nasal potential difference; TER: Transepithelial electrical resistance; WT: Wild type.

## Competing interests

S.M.R served as PI for CF Clinical Trials sponsored by Vertex Pharmaceuticals and Novartis. He has received COPD-related grant funding from NHLBI and Forest Research Institute. M.T.D has served on COPD-related advisory boards for Ikaria, Forest, GlaxoSmithKline and Boehringer Ingelheim. He has served as site PI for contracted COPD clinical trials sponsored by Aeris, Centocor, Pfizer, Otsuka, Pulmonx, GlaxoSmithKline and Boehringer Ingelheim. He has received COPD-related grant funding from Forest, GlaxoSmithKline and NHLBI.

## Authors’ contributions

SVR, JHT, MTD, and SMR conceived of the experiments; SVR, JHT, and SKGP, conducted the research; SVR, JHT, RO, PF, MTD, MTD, and SMR analyzed the data; SVR and SMR wrote the manuscript; SMR and MTD supervised the project. All authors read and approve the final manuscript.
